# Retraction: Parthenolide Augments the Chemosensitivity of Non-small-Cell Lung Cancer to Cisplatin *via* the PI3K/AKT Signaling Pathway

**DOI:** 10.3389/fcell.2021.696217

**Published:** 2021-05-05

**Authors:** Frontiers Editorial Office

Following publication, concerns were raised regarding image manipulation. The authors failed to provide a satisfactory explanation during the investigation, which was conducted in accordance with Frontiers' guidelines.

This retraction was approved by the Chief Editors of Frontiers in Cellular and Development and the Editor-in-Chief of Frontiers. The authors [did] agree to this retraction.

